# Primary Cutaneous CD30-Positive Large T-Cell Lymphoma in an 80-Year-Old Man: A Case Report

**DOI:** 10.5402/2011/634042

**Published:** 2011-03-30

**Authors:** Rehan Hussain, Amir Bajoghli

**Affiliations:** ^1^School of Medicine, The George Washington University, Washington, DC 20037, USA; ^2^Skin and Laser Surgery Center, Center for Mohs & Skin Cancer Surgery, 8130 Boone Boulevard No. 340, Vienna, VA 22182, USA

## Abstract

Primary cutaneous CD30-positive large cell lymphoma (CD30+ PCLCL) is a rare subtype of cutaneous T-cell lymphoma (CTCL) that can present in a variety of ways. We report a patient with a three-month history of an enlarging, exophytic mass with two smaller satellite lesions on the left forearm. Biopsy of the skin stained positive for CD30, and, after thorough systemic evaluation, a diagnosis of CD30+ PCLCL was made. When PCLCL is suspected, it is important to perform immunohistological studies for CD30 types and conduct a thorough workup to rule out systemic LCL. These measures will reduce the use of unnecessarily aggressive chemotherapy regimens for CD30+ PCLCL, an indolent disease with a favorable prognosis.

## 1. Introduction

CD30+ PCLCL represents about ten percent of all cases of cutaneous T-cell lymphomas [1], and it is thought to represent a spectrum of disease, with lymphomatoid papulosis at the benign end, and primary cutaneous anaplastic large cell lymphoma (PCALCL) at the other [2, 3]. CD30-positive large cell lymphomas are typically associated with poor prognosis when they are systemic, though they have a favorable prognosis when confined to the skin. CD30 expression is a much more important prognostic parameter than is the extent of skin disease at presentation, as Beljaards et al. [4] described that 80% of patients with CD30-negative PCLCL died of progressive disease (median 27 months after diagnosis) compared to only 7% of patients with CD30+ PCLCL.

## 2. Case Presentation

**Figure 1:**
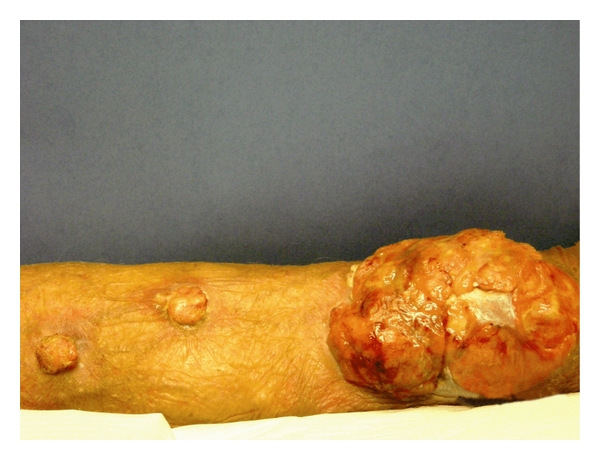
Patient's left forearm with large exophytic tumor and two satellite tumors.

**Figure 2:**
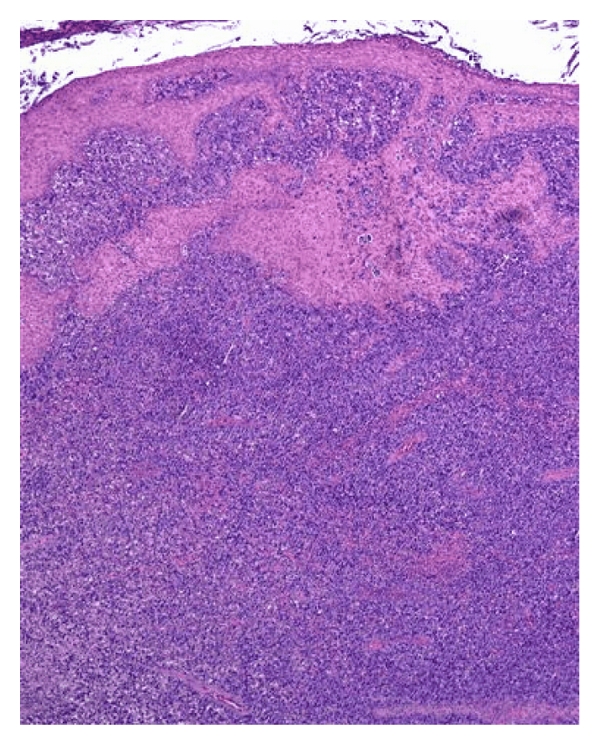
H&E staining of the tumor shows a dense perivascular and band-like monomorphous infiltrate composed of markedly atypical, large, pleomorphic epithelioid cells, along with immature cells resembling immunoblasts.

**Figure 3:**
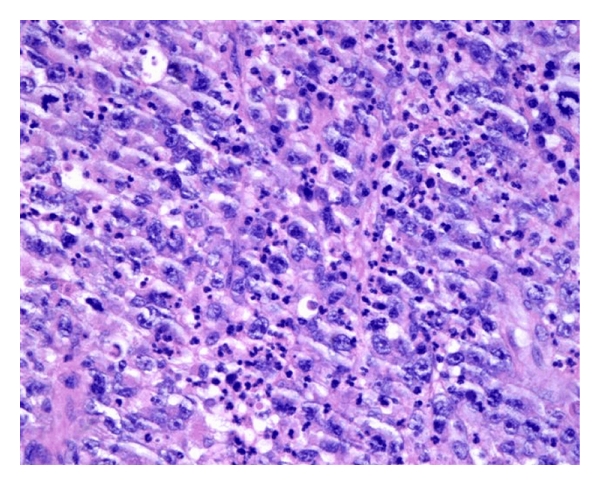
H&E staining of the tumor shows a dense perivascular and band-like monomorphous infiltrate composed of markedly atypical, large, pleomorphic epithelioid cells, along with immature cells resembling immunoblasts.

**Figure 4:**
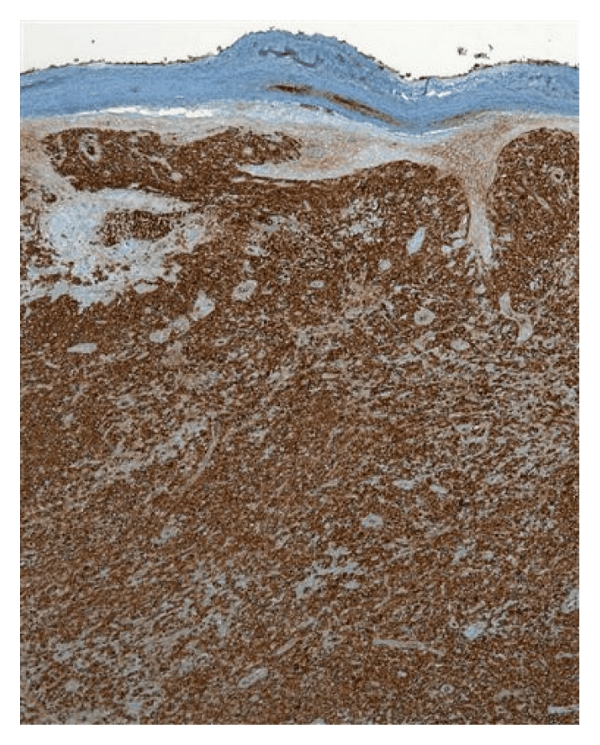
Histological image of the tumor stained to show expression of the CD30 surface marker on the atypical lymphoid cells.

**Figure 5:**
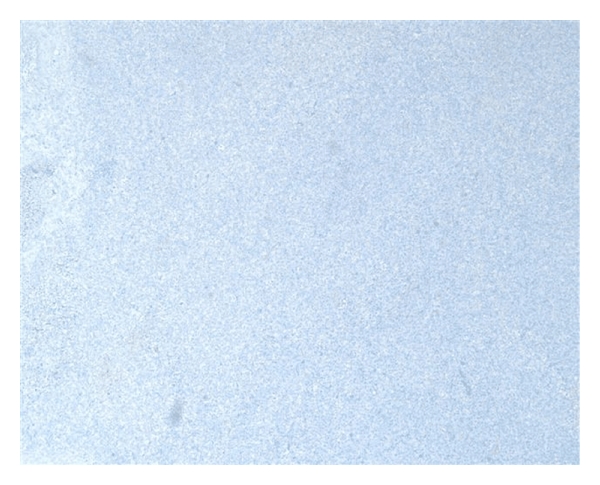
Histological image of the tumor that shows absence of the ALK-1 protein on the atypical lymphoid cells.

**Figure 6:**
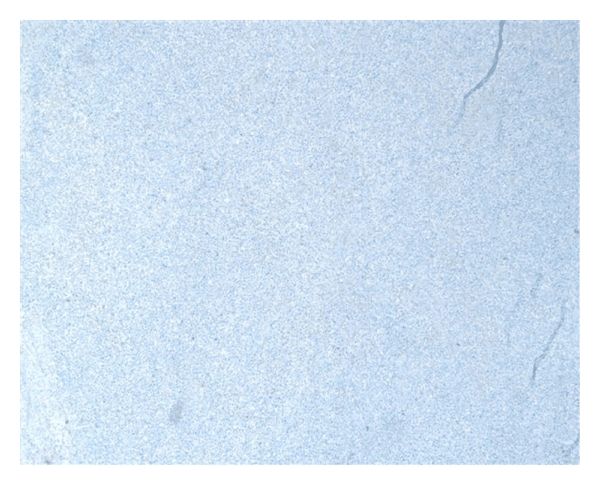
Histological image of the tumor that shows absence of the EMA surface marker on the atypical lymphoid cells.

An 80-year-old male was seen with a three-month history of a mass on the left inner forearm that was getting persistently larger and bleeding. Physical examination showed the presence of a 10.0 × 7.0 cm exophytic nodule on the left wrist and forearm. Two smaller satellite nodules were seen along the mid dorsal forearm, each measuring 2.0 × 2.0 cm (Figure 1). No adenopathy was noted. CBC and other laboratory values were unremarkable. A bone marrow biopsy was performed, and the results were normal. PET scan, CT scan of the chest and abdomen, and chest X-ray were all normal. A biopsy was taken at the time. It showed a polypoid lesion characterized by a dense perivascular and band-like monomorphous infiltrate composed of markedly atypical, large, pleomorphic epithelioid cells, as well as immature cells resembling immunoblasts (Figures 2 and 3). Mitoses and tumor necrosis with accompanying neutrophils were noted. Reed-Sternberg cells, however, were not observed. Over 75% of the atypical lymphoid cells expressed CD30 (Figure 4), but not ALK1 (Figure 5) and EMA (Figure 6), which led to the diagnosis of CD30+ primary cutaneous anaplastic large cell lymphoma (CD30+ PCLCL). The patient received localized radiation treatment with excellent results, as the tumor has completely remitted.

## 3. Discussion

CD30+ PCLCL usually presents in adults, age 45–60, and is six-times more frequent in males [5]. It presents as one to several localized nodules or tumors with ulceration. Twenty percent of cases are multifocal, and the trunk and extremities are most commonly involved. Plaques are greater than 1 cm in most cases (77%). Draining lymph nodes are a positive finding in about 25% of cases. Additional common features are epidermal ulceration (63%), prominent vascular proliferation (60%), pseudoepitheliomatous hyperplasia (55%), tumor necrosis (55%), and vascular infiltration by neoplastic cells (44%) [5]. In 20–25% of cases, Reed-Sternberg-like pleomorphic or immunoblastic cells are present [6]. Presentation may be variable, with this lesion being mistaken for other skin disorders, such as adult-onset eczema, pyoderma gangrenosum, morphea, localized scleroderma, or squamous cell carcinoma [7]. 

Histopathologically, dense clusters or nodules of large CD30+ tumor cells are observed in PCALCL; more than 75 percent of tumor cells should be CD30+ for a diagnosis of PCALCL. The CD30+ tumor cells are CD4+, and can have loss of T-cell markers, such as CD2, CD3, and CD5. Cell surface markers can help in distinguishing PCALCL from its primary nodal counterpart with secondary cutaneous disease. The PCALCL tumors more commonly express HECA-452 and not EMA whereas secondary disease is more likely to express EMA and not HECA-452 [1]. Systemic lymphomas are also more likely to contain the *t*(2;5) translocation, which creates the fusion protein NPM-ALK (nucleophosmin-anaplastic lymphoma kinase) [8, 9]. 

CD30+ anaplastic large cell lymphomas are typically associated with poor prognosis when they are systemic, though they have a favorable prognosis when confined to the skin [4]. The survival rate of CD30+ PCLCL is 95% at five years after diagnosis, and about 20% of lesions regress spontaneously [10]. However, CD30-negative PCLCL is a much more aggressive neoplasm (though it is indistinguishable from CD30+ PCLCL on gross examination), with a 15% 5-year survival [4]. It is crucial to distinguish CD30+ PCLCL from primary nodal LCL with secondary cutaneous involvement, because patients with secondary skin disease generally have a worse prognosis and need to be treated more aggressively. The presence of draining lymph nodes does not seem to alter prognosis. There are no clinical differences in presentation, course, or prognosis between anaplastic and nonanaplastic CD30+ LCL [4]. 

The standard treatment of single or localized PCALCL lesions is either local excision or radiation. Chemotherapy is usually reserved for patients with systemic involvement. The treatment of patients with only skin and nodal disease is controversial, with some practitioners favoring radiation and chemotherapy for these patients [1].

## 4. Conclusion

Primary cutaneous CD30-positive large T-cell lymphoma may present in a variety of ways, but is definitively distinguished from other cutaneous T-cell lymphomas by the expression of the CD30 antigen on immunohistological staining. Other cell surface markers, such as EMA and ALK1 are helpful in distinguishing CD30+ PCLCL from secondary cutaneous CD30+ LCL, which influences the prognosis and decision to add chemotherapy to the treatment regimen.
